# Targeted therapy or immunotherapy in BRAF-mutated metastatic melanoma: a Spanish center’s decade of experience

**DOI:** 10.3389/fonc.2024.1322116

**Published:** 2024-02-21

**Authors:** Chen Sun, Sofia España, Nina Richarz, Carme Solé-Blanch, Aram Boada, Anna Martinez-Cardús, Alan Chu, Zongwen Liu, Jose Luis Manzano

**Affiliations:** ^1^ Department of Radiation Oncology, The Second Affiliated Hospital of Zhengzhou University, Zhengzhou, China; ^2^ Medical Oncology Department, Institut Catala d´Oncologia Badalona, Universitari Hospital Germans Trias i Pujol, Badalona-Applied Research Group in Oncology (B-ARGO), Germans Trias i Pujol Research Institute (IGTP), Badalona, Spain; ^3^ Dermatology Department, Universitari Hospital Germans Trias i Pujol, Badalona, Spain; ^4^ Badalona-Applied Research Group in Oncology (BARGO), Germans Trias i Pujol Research Institute (IGTP), Badalona, Spain; ^5^ Dermatology Department, Universitari Hospital Germans Trias i Pujol, Germans Trias i Pujol Research Institute, Autonoma University of Barcelona, Badalona, Spain

**Keywords:** target therapy, immunotherapy, melanoma, BRAF mutation V600, clinical experience

## Abstract

**Background:**

Targeted therapies and immunotherapy are currently considered the mainstay first-line treatment for advanced BRAF-mutated melanoma. However, the impact of treatment (targeted therapy and immunotherapy) and the prognostic factors are still not clear.

**Material and methods:**

Medical records of 140 patients diagnosed with advanced melanoma between 2011 and 2021 were retrospectively reviewed to extract demographic, BRAF status, treatment, performance status, and survival data. ORR, PFS, and OS were compared between patients diagnosed with advanced melanoma and treated with first-line IT or BRAF/MEKi. The prognostic factors were assessed using Cox regression models.

**Results:**

In all patients and those treated with immunotherapy, we did not find any effect of BRAF status on ORR, PFS, or OS. In patients with BRAF-mutated melanoma, ORR was 43.8% vs. 70% (P=0.04), PFS was 19.2 vs. 11.5 months (p=0.22), and OS was 33.4 vs. 16.4 months for the immunotherapy and targeted therapy groups, respectively (P=0.04). ECOG, presence of brain metastases, and high LDH level from initiation of first-line treatment were all associated with differences in PFS and OS.

**Conclusion:**

Patients with advanced BRAF-mutated melanoma treated with first-line immunotherapy had a significantly longer PFS and OS than those treated with first-line BRAF/MEKi; however, first-line BRAF/MEKi treatment had a significantly higher ORR than first-line immunotherapy.

## Introduction

The prognosis of advanced melanoma has radically changed over the last decade with the incorporation of targeted therapies and immunotherapy (1).

On the one hand, we have three combinations of targeted therapies (TT) with BRAF-MEK inhibitors (dabrafenib-trametinib, vemurafenib-cobimetinib, and encorafenib-binimetinib) in patients with a BRAF mutation (50% of all subtypes of cutaneous melanoma). These combinations have demonstrated a high response rate and benefit in progression-free survival (PFS) and overall survival (OS) with respect to monotherapy treatment with the BRAF inhibitor, for which they have been approved by both the U.S. Food and Drug Administration (FDA) and the European Medicines Agency (EMA). The three combinations present similar results as regards efficacy, differing in their toxicity profiles (2–4).

And, in parallel, we have immunotherapy (IT), more specifically immune checkpoint inhibitors (anti-PD-1 and anti-CTL-4), represented by pembrolizumab, nivolumab, and the combination of nivolumab and ipilimumab (NIV/IPI), which have shown consistent benefits in patients with advanced melanoma, both in BRAF-mutated and wild-type populations (5, 6).

*BRAF* is a proto-oncogene belonging to the RAF family of serine-threonine protein kinases; 50% of patients with cutaneous melanoma have *BRAF* mutations, with the glutamic acid for valine substitution at position 600 (V600E) representing about 90% of all *BRAF* mutations. BRAF-mutated melanoma presents different clinical features and a more aggressive biological behavior, with a greater tendency to present distant metastases and brain lesions (7).

With all the above, the first-line treatment for metastatic melanoma is immunotherapy or targeted therapy. TT provides high clinical responses which are usually transient due to the appearance of resistance mechanisms; IT has lower responses but a longer response duration than targeted therapy.

In clinical practice, having two types of effective therapies available and no biomarkers to select one treatment over the other, the choice is based solely on the characteristics of the patient (age, comorbidities) and the disease (location of metastases, number of metastatic sites).

Two randomized studies (SECOMBIT, DREAM-SEQ) (8, 9) analyzing treatment sequences in advanced BRAF-mutated melanoma have recently published their data. Pending more mature follow-up data about survival, they support the use of immunotherapy (nivolumab and ipilimumab) as the first-line sequence rather than targeted therapy.

Our work on retrospective characterizations aimed to study the impact of treatment sequences (response rate, PFS, and OS), as well as to identify prognostic factors that could help select the best treatment option in advanced melanoma using real-world data collected from routine clinical practice.

## Materials and methods

### Patients

Data were retrospectively collected from all patients with advanced melanoma (stage IV and III non-resectable) treated with BRAF/MEKi or IT as first-line treatment at the Catalan Institute of Oncology, Badalona, Hospital Universitari Germans Trias i Pujol from January 2011 to March 2021. Clinical data were obtained from medical records and included age, gender, Eastern Cooperative Oncology Group (ECOG) performance status at diagnosis, number of metastases appearing during first-line treatment, brain metastases, liver metastases, LDH (lactate dehydrogenase) level from initiation of first-line treatment, and second‐line therapy received. All data was anonymized.

Inclusion criteria were confirmed diagnosis of advanced melanoma, age ≥18 years, and ECOG performance status (PS) ≤2; patients were treatment-naive and had received first-line IT or BRAF/MEKi after the initial diagnosis of advanced melanoma. BRAF mutated patients only included patients with BRAFV600E mutants and did not include patients with other mutation types. Patients with pre-existing brain or liver metastases were asymptomatic or required to have undergone either surgery or stereotactic radiosurgery (SRS) before treatment assignment and had no signs of disease progression. Patients were excluded if they had another primary tumor, severe infections or gastrointestinal bleeding, cerebral hemorrhage, cerebral infarction, or mental illness. Patients with incomplete clinical data or insufficient follow‐up (less than 30 days) from initiation of first‐line therapy were also excluded.

As part of normal clinical practice, *RAS* and *BRAF* mutations were determined in all patients by polymerase chain reaction (PCR) or pyrosequencing (10, 11), as previously described.

Patients were subdivided according to first-line treatment: TT and IT. Patients in the TT group were treated with BRAF/MEKi and in the IT group with NIV/IPI or anti-PD1. The OS and objective response rate (ORR) were calculated for all patients and subgroups.

**Figure 1:**
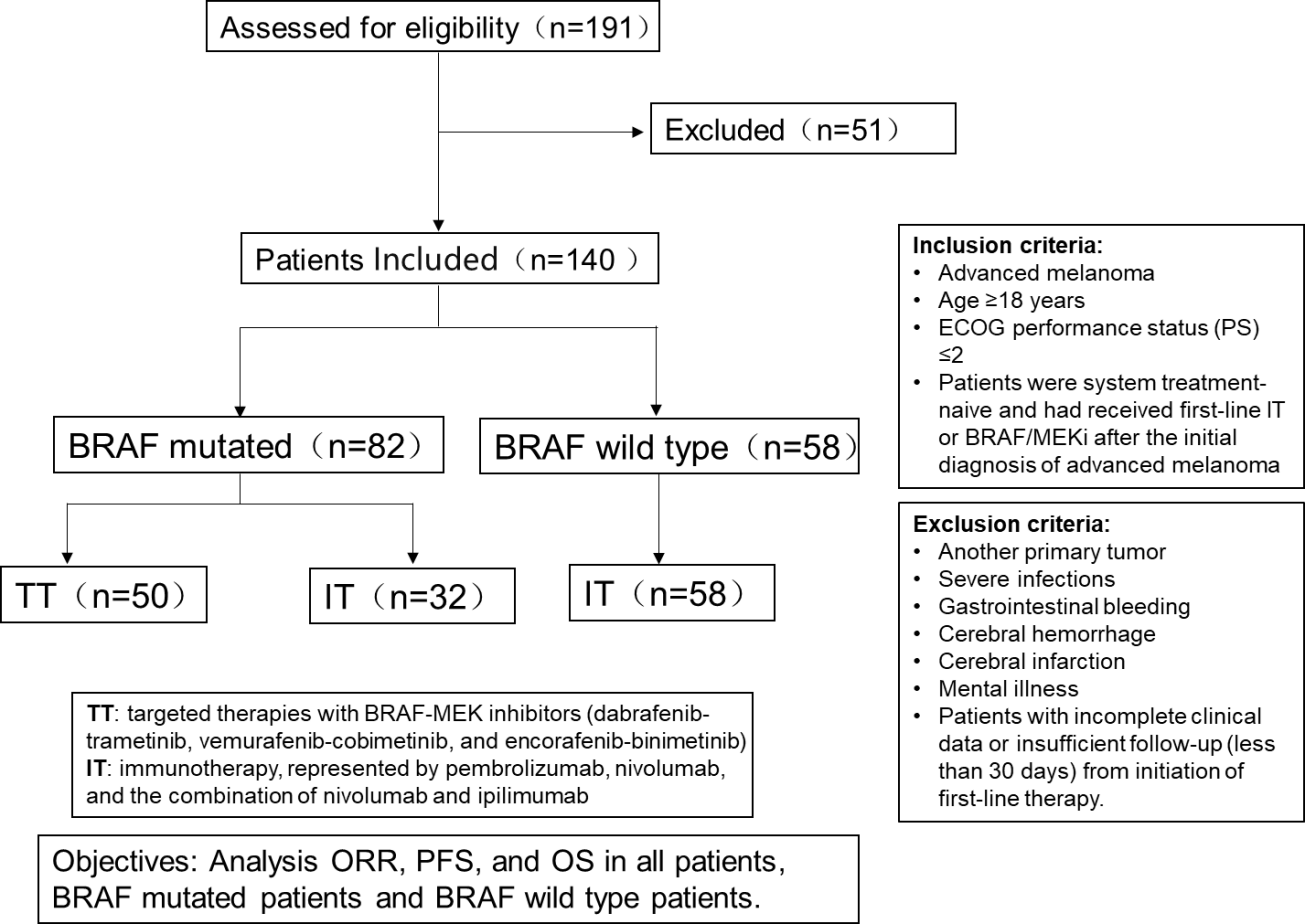
The flowchart of the study.

A flowchart of this study is shown in **Figure 1**.

The study was approved by the Clinical Research Ethics Committee of the hospital and all patients gave their signed informed consent. The study was carried out following the hospital guidelines and those of the Declaration of Helsinki and its amendments.

### Statistical analyses

Nominal variables were analyzed using the Chi-square or Fisher’s exact test as appropriate, and quantitative variables were analyzed using the student’s t-test. Response to treatment was classified according to the Response Evaluation Criteria in Solid Tumors (RECIST 1.1). The ORR was defined as the proportion of patients attaining a complete or partial response. The disease control rate (DCR) was defined as the proportion of patients attaining a complete or partial response or stable disease. On the basis of the best overall response, patients with complete or partial response were considered responders, while others with stable or progressive disease were considered non_responders.OS was calculated from the time of diagnosis of advanced melanoma until death from any cause or final follow-up. PFS was calculated from the time of diagnosis of advanced melanoma until disease progression. Patients with no progression or death at the time of analysis were censored. Median OS and PFS, with 95% confidence intervals (CI), were calculated via the Kaplan-Meier method and compared with the log-rank test. In order to determine the most relevant prognostic factors in our population, OS was calculated for different variables, including gender, age, first-line ECOG, number of metastases appearing during first-line treatment, brain metastases, liver metastases, LDH level, and second‐line therapy received. Univariate analysis was performed to identify significant prognostic factors of OS and PFS (p<0.1). Variables found to have a significant impact on OS and PFS were included in the multivariate Cox regression model. Significance was set at p ≤ 0.05. All reported p-values were two-sided. Statistical analyses were performed using SPSS version 24 (IBM).

## Results

### Patients’ characteristics

A total of 140 patients with advanced melanoma were included in the study. The median age was 63.0 years (range, 24-78). There were more males than females (74 males vs. 66 females). Baseline characteristics were well balanced between BRAF-mutated (*n* = 82) and BRAF-wild type patients (*n* = 58), including ECOG, number of metastases appearing during first-line treatment, appearance of brain and liver metastases, LDH level, second‐line therapy received. There were 53 patients with pre-existing brain or liver metastases, 32 patients with brain metastases and 21 were with liver metastases. There were 67 patients that developed (e.g., brain, liver, other) metastases after first-line treatment. There were ≥2 metastatic sites in 36.4% of patients, and 22.9% had CNS (Central Nervous System) metastasis (**Table 1**). However, a significantly higher incidence of brain metastases was noted in patients of the BRAF-mutated group (30.5%) when compared with BRAF-wild type (12.1%) (p=0.02).

**Table 1:** Baseline and patients’ characteristics.

	All Patients N=140 N (%)	BRAF-mutated N=82 N (%)	BRAF-wild type N=58 N (%)	p* value
**Age (years)**				0.23
Median, range	63.0 ± 14.6	63.0 ± 14.1	66.0 ± 15.0	
**Gender**				0.11
Male	74 (52.9)	48(58.5)	26(44.8)	
Female	66 (47.1)	34(41.5)	32(55.2)	
**ECOG**				0.17
PS 0-1	120 (85.7)	72(83.7)	51(87.9)	
PS 2	20 (14.3)	14(16.3)	7(12.1)	
**Number of metastasis during first-line treatment**				0.20
1	89 (63.6)	52(63.4)	37(63.8)	
2	34 (24.3)	17(20.7)	17(29.3)	
>2	17 (12.1)	13(15.9)	4(6.9)	
**Brain metastases**				0.02
Yes	32 (22.9)	25(30.5)	7(12.1)	
No	108 (77.1)	57(69.5)	51(87.9)	
**Liver metastases**				0.34
Yes	21 (15.0)	10(12.2)	11(19.0)	
No	119 (85.0)	72(87.8)	47(81.0)	
**LDH level**				0.94
Normal	64 (45.7)	37(45.1)	27(46.6)	
Elevated	53 (37.9)	32(39.0)	21(36.2)	
Unknown	23 (16.4)	13(15.9)	10(17.2)	
**Second‐line therapy received**				0.73
Yes	56 (40.0)	34 (41.5)	22 (37.9)	
No	84 (60.0)	48 (58.5)	36 (62.1)	

Fifty patients received TT (median number of cycles, 14.6; range, 4-68) and 90 received IT (median number of cycles, 15.7; range, 4-76). The BRAF-mutated (BRAFm) group consisted of 82 patients: 50 were treated with TT and 32 with IT. The BRAF-wild type (BRAFwt) group consisted of 58 patients which all treated with IT. Among all 90 patients treated with IT, 28 patients received Nivolumab, 36 patients received Pembrolizumab, and 36 patients received Nivolumab + Ipilimumab. Fifty patients were responders to the IT, and 40 patients were non-responders. Patients without IT or TT were not included in the survival analyses of the BRAFm or BRAFwt groups. All patients received treatment until progression or intolerable toxicity. Median follow-up was 28.4 months (range 18.2-36.3).

### ORR & PFS, and OS

**Tables 2A–D** presents the ORR values for all patients and each subgroup. ORR was 63.4% for the BRAFm subgroup and 54.8% for BRAFwt. ORR was not significantly different between the BRAFm and BRAFwt subgroups (p = 0.42). Among BRAFm patients, those treated with TT demonstrated a better ORR than patients treated with IT (70.0% vs. 43.8%, p=0.04). Of the patients treated with IT, the ORR was not significantly different between the BRAFm and BRAFwt subgroups (p=0.80),and the ORR between combination therapy and monotherapy was not significantly different either (p=0.93).

**Table 2:** Objective response to treatment.

	CR	PR	SD	PD	ORR	Total	P value
(A) ORR of BRAFm and BRAFwt patients							
**BRAF-mutated**	12 (14.6%)	37 (45.1)	11 (13.4)	22 (26.8)	52 (63.4)	82	0.42
**BRAF-wt**	3 (7.9%)	14 (42.1)	6 (15.8)	15 (39.5)	31 (54.8)	38	
**total**	15	51	17	37	84	120	
(B) ORR of BRAFm patients treated with immunotherapy and targeted therapy							
**IT**	4 (12.5%)	10 (31.3%)	7 (21.9%)	11 (34.4%)	14(43.8)	32	**0.04**
**TT**	8 (16.0%)	27 (54.0%)	4 (8.0%)	11 (22.0%)	35 (70.0)	50	
**total**	12	37	11	22	49	82	
(C) ORR of BRAFm and BRAFwt patients treated with immunotherapy							
**BRAF-mutated**	4 (12.5)	10 (31.3)	7 (21.9)	11 (34.4)	14 (43.8)	32	0.80
**BRAF-wt**	8 (13.8)	19 (32.8)	11 (19.0)	20 (34.5)	27 (46.6)	58	
**total**	12	29	18	31	41	90	
(D) ORR of patients treated with immunotherapy that received combination therapy and monotherapy							
**Combination therapy**	12 (18.8%)	24 (37.5%)	7 (10.9%)	21 (32.8%)	36(56.3%)	64	0.93
**Monotherapy**	4(15.4%)	10(38.5%)	4(15.4%)	8(30.8%)	14(53.8%)	26	
**total**	16	34	11	29	31	90	

CR, complete response; PR, partial response; SD, stable disease; PD, progressive disease; IT, immunotherapy; TT, targeted therapy. The bold value is a significative value in the analysis.

**Figure 2:**
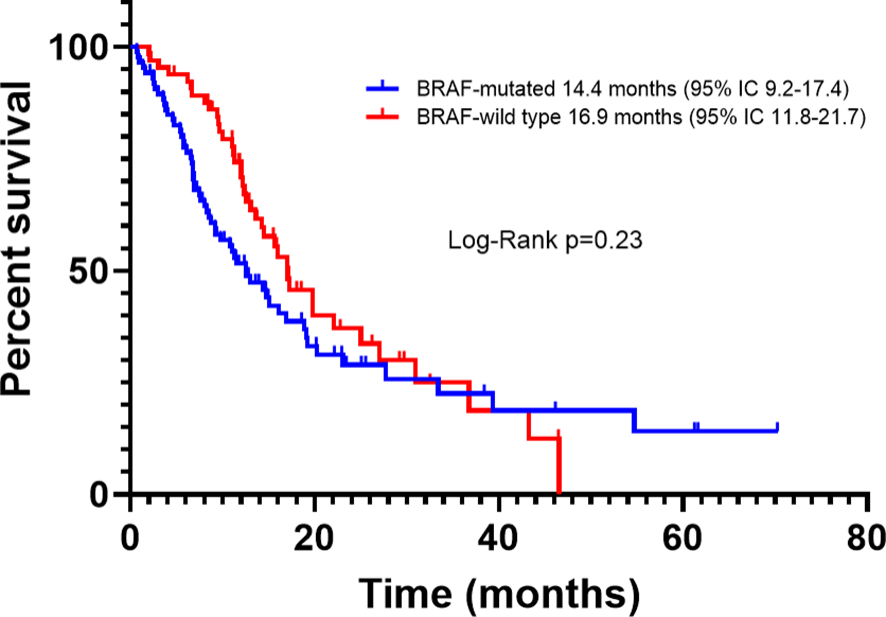
Progression-free survival in advanced BRAF-mutated and BRAF-wild type melanoma patients.

**Figure 3:**
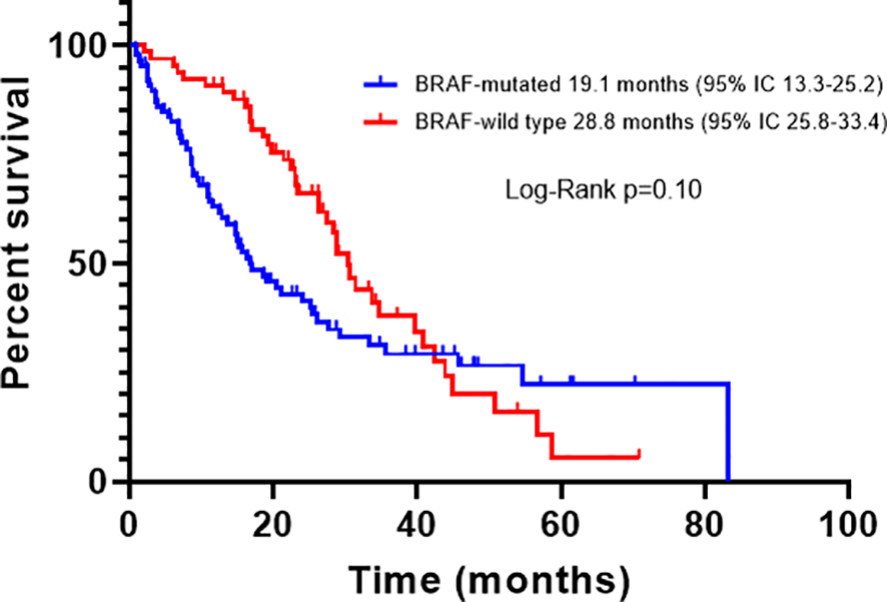
Overall survival in advanced BRAF-mutated and BRAF-wild type melanoma patients.

**Figure 4:**
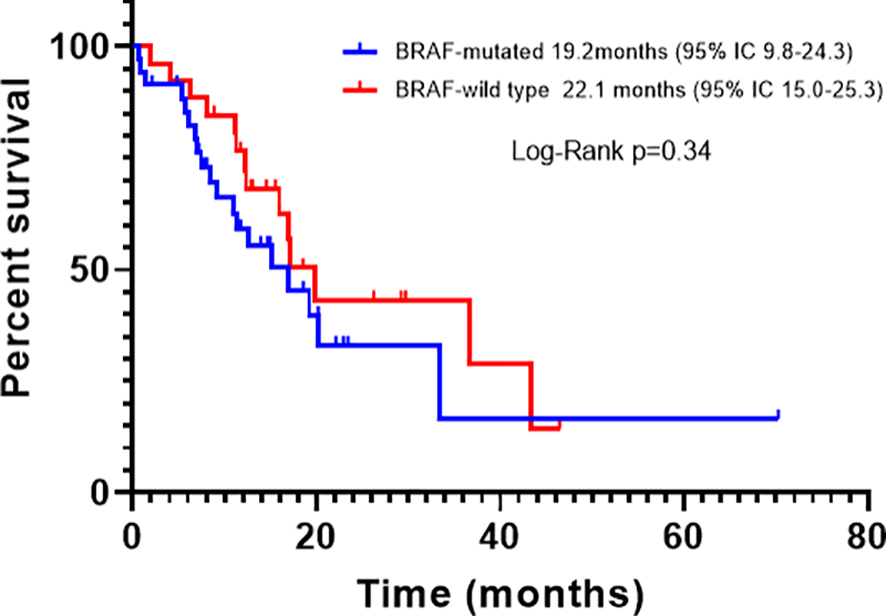
Progression-free survival in advanced BRAF-mutated and BRAF-wild type melanoma patients treated with first-line immunotherapy.

**Figure 5:**
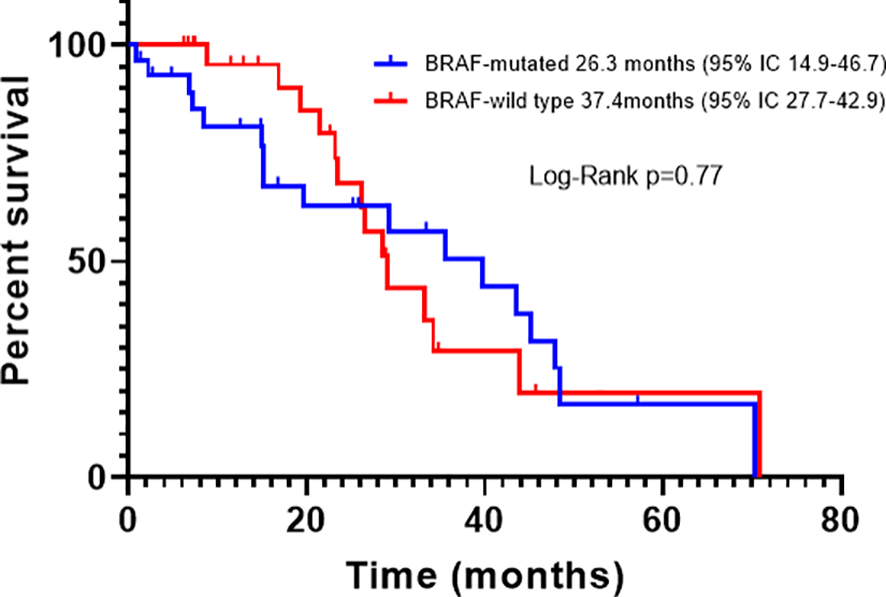
Overall survival in advanced BRAF-mutated or BRAF-wild type melanoma patients treated with first-line immunotherapy.

For all patients, PFS was 16.1 months (95% CI, 12.0-20.1); according to BRAF status, the PFS of BRAFm patients was 14.4 months (95% CI, 9.2-17.4), and of BRAFwt it was 16.9 months (95% CI, 11.8-21.7). There was a trend toward improved PFS in BRAFwt patients compared with BRAFm, however, it did not reach statistical significance (p=0.23) (**Figure 2**). For all patients, the median OS was 29.3 months (95% CI, 18.1-39.4). The OS also was higher for the BRAFwt group (28.8 vs. 19.1 m, p=0.10) (**Figure 3**). If we compared both groups treated with IT, the PFS of BRAFm patients was 19.2 months (95% IC 9.8-24.3) and of BRAFwt 22.1 months (95% IC 15.0-25.3) (**Figure 4**). This benefit was maintained for OS in the BRAFwt group (BRAFwt 37.4 months vs. BRAFm 26.3 months, p=0.77) (**Figure 5**).

**Figure 6:**
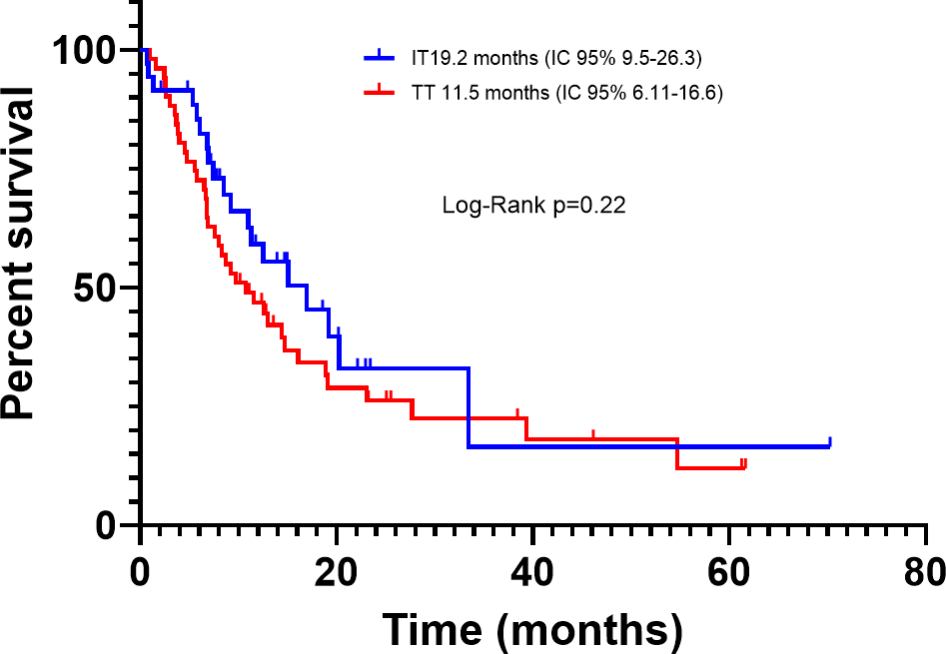
Progression-free survival in advanced BRAF-mutated melanoma patients treated with immunotherapy or targeted therapy.

**Figure 7:**
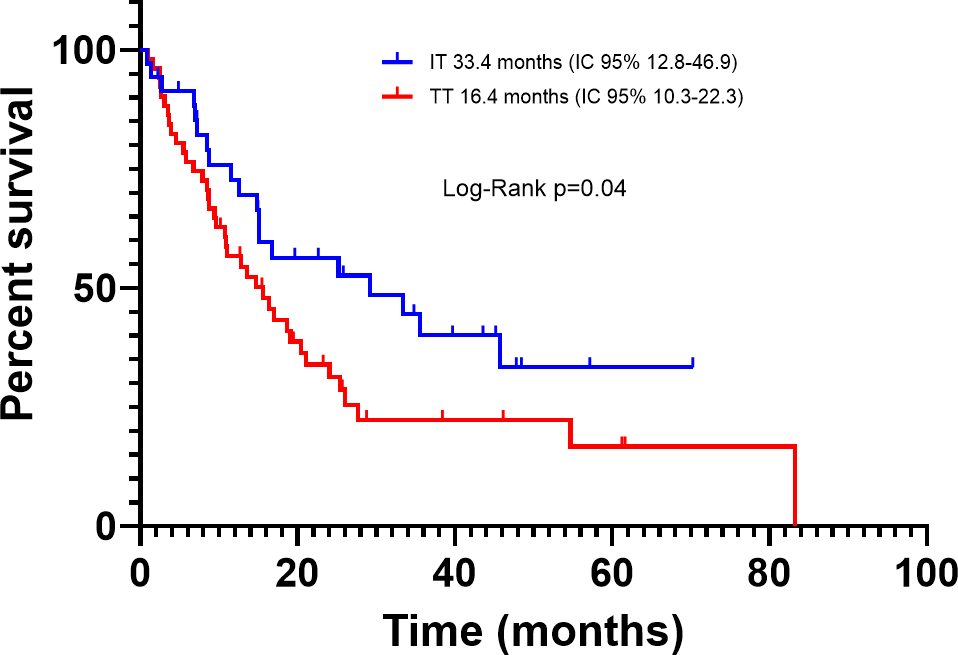
Overall survival in advanced BRAF-mutated melanoma patients treated with first-line immunotherapy or targeted therapy.

Among the BRAFm subgroup, according to treatment, the median PFS was 11.5 months (95% CI, 6.11-16.6) and 19.2 months (95% CI, 9.5-26.3) for TT and IT, respectively (P=0.22) (**Figure 6**). The PFS hazard ratio for death for TT vs. IT was 1.66 (95% CI 0.99–2.64; p=0.05) and the median OS was 16.4 months (95% CI, 10.3-22.3) and 33.4 months (95% CI, 12.8-46.9), respectively (p=0.04) (**Figure 7**). OS was significantly longer in patients treated with IT compared with TT. The OS hazard ratio for death for TT vs. IT was 1.83 (95% CI 1.12–2.95; p=0.01).

**Figure 8:**
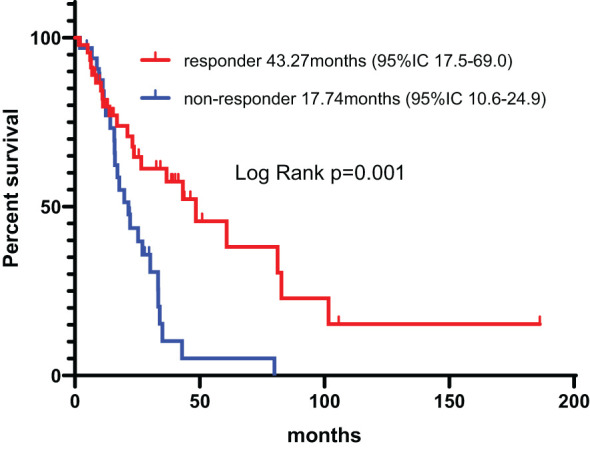
Progression-free survival in patient responders and non-responders to the IT.

**Figure 9:**
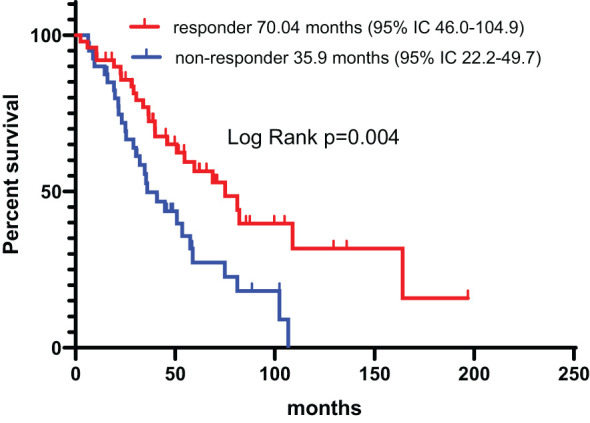
Overall survival in patient responders and non-responders to the IT.

Among the patients treated with IT, according to the treatment strategies, the median PFS was 22.1 months (95% CI, 11.9-32.2) and 19.8 months (95% CI,9.4-30.1) for combination therapy and monotherapy, respectively (p=0.74), and the OS was 57.5 months (95% CI, 39.9-67.3) and 53.52 months (95% CI, 34.7-80.3), respectively (p=0.61). According to the response of the IT, the median PFS was 43.3 months (95% CI, 17.5-69.0) and 17.7 months (95% CI, 10.6-24.9) for responder and non-responders to the IT, respectively (p=0.001) (**Figure 8**), and the OS was 75.0 months (95% CI, 46.0-104.9) and 35.9 months (95% CI, 22.2-49.7), respectively (p=0.004) (**Figure 9**). The PFS and OS was significantly longer in patient responders to IT.

Among all patients, 36.6% (30/82) of the BRAFm subgroup and 45.8% (21/58) of the BRAFwt subgroup were alive at the end of the study. Of the patients only treated with first-line treatment, 45.8% (22/48) of BRAFm and 50.0% (18/36) of BRAFwt patients were alive at the end of this study. All the patients who died was due to melanoma.

Among the BRAFm patients treated with TT, OS rates were 62.2% (95% CI, 50.24% to 74.16%) at one year, 40.2% (95% CI, 27.46% to 52.94%) at two years, 20.6% (95% CI, 8.84% to 32.36%) at three years, and 17.2% (95% CI, 5.64% to 28.76%) at four years; among those treated with IT, the OS rates were 79.9% (95% CI, 68.14% to 91.66%) at one year, 52.9% (95% CI, 37.61% to 68.19%) at two years, 41.5% (95% CI, 29.52% to 60.48%) at three years, and 32.2% (95% CI, 15.74% to 48.66%) at four years.

The PFS rates for the TT group were 41.3% (95% CI, 28.76% to 53.84%) at one year and 6.2% (95% CI, 0 to 16.39%) at two years; the PFS rates for the IT group were 53.1% (95% CI, 37.81% to 68.38%) at one year and 27.5% (95% CI, 10.06% to 44.94%) at two years.

### Prognostic factors

In the univariate analysis, the factor associated with PFS and OS resulting in a lower OS were being over 65 years of age, ECOG PS≥ 2, brain metastases, liver metastases, and high LDH level (p<0.1). The prognostic factors resulting in a lower PFS were being over 65 years of age, ECOG PS≥ 2, >2 metastatic sites appearing during first-line treatment, brain metastases, liver metastases, high LDH level, and first-line treatment with IT. (p<0.1). The appearance of >2 metastatic sites during first-line treatment (HR 1.26 95% CI 1.06‐2.49, p=0.04) was associated with decreased OS, however, this did not reflect on PFS.

Multivariate Cox regression analysis confirmed that these variables were associated with PFS and OS (**Tables 3A**, **B**). After adjusting for other prognostic factors, multivariate Cox regression analyses confirmed that ECOG PS≥ 2, presence of brain metastases, and high LDH level from initiation of first-line treatment were all associated with differences in PFS and OS.

**Table 3A:** Univariate cox regression of all patients and variables effect on progression-free survival and overall survival.

Variable	PFS		OS	
	HR (95%CI)	*P*	HR (95%CI)	*P*
**Age (**≥65 vs <65)	1.91(0.85-3.02)	0.04	1.67(0.81-1.93)	0.03
**Gender (**Male vs Female)	1.02(0.67-1.55)	0.94	1.01(0.63-1.64)	0.93
**ECOG (**≥2 vs 0-1)	2.15(1.95-3.08)	0.02	2.43(1.87-3.34)	0.03
**BRAF status (**BRAF wt vs BRAF mutated)	0.85(0.55-1.30)	0.45	0.79(0.51-1.21)	0.28
**Number of metastasis during first-line treatmen**t(≥2 vs 1)	2.11(0.97-4.14)	0.21	1.98(1.09-3.62)	0.03
**Brain metastases** (Yes vs No)	2.01(1.26-3.21)	<0.01	2.07(1.29-3.32)	<0.01
**Liver metastases** (Yes vs No)	1.55(0.90-2.68)	0.03	1.42(0.82-2.46)	0.08
**LDH level** (Normal vs Elevated)	0.60(0.26-1.42)	0.03	0.44(0.19-1.03)	0.06
**Second-line therapy received** (Yes vs No)	1.51(0.97-2.30)	0.14	1.31(0.87-2.01)	0.20

**Table 3B:** Multivariate cox regression of all patients and variables effect on progression-free survival and overall survival.

Variable	PFS		OS	
	HR (95%CI)	*P*	HR (95%CI)	*P*
**Age (**≥65 vs <65)	1.34(0.91-2.18)	0.36	1.28(0.63-2.11)	0.31
**ECOG (**≥2 vs 0-1)	2.01(1.99-3.13)	0.02	2.58(1.38-3.54)	<0.01
**BRAF status (**BRAF wt vs BRAF mutated)	0.73(0.41-1.30)	0.29	0.73(0.51-1.76)	0.36
**Number of metastases during first-line treatment** (≥2 vs 1)	2.09(0.98-4.41)	0.09	–	–
**Brain metastases** (Yes vs No)	2.14(1.28-3.56)	0.02	1.78(1.25-3.33)	<0.01
**Liver metastases** (Yes vs No)	1.34(0.63-2.85)	0.45	1.62(0.92-2.83)	0.26
**LDH level** (Normal vs Elevated)	0.62(0.28-0.89)	0.04	0.55(0.37-0.88)	0.03

## Discussion

In our retrospective analysis, patients with advanced BRAF-mutated melanoma treated with first-line IT treatment displayed significant OS benefits compared with those treated with TT (BRAF plus MEK inhibitors). Regarding patients who only received first-line treatment, more patients remained alive in the IT than in the TT subgroup.

These results were similar to other analyses published previously. The Checkmate 067 clinical trial reported a median OS of 72.1 months with NIV/IPI and, in our study, the median OS of the IT subgroup was 37.4 months (12). The COMBI-d trial reported a median OS with dabrafenib plus trametinib (D+T) of 24 months (2); in our study, the median OS was 17 months, but in a real-world experience study (ADMIRE), the median OS for patients treated with first‐line BRAF/MEKi was 15.4 months (13). In our sample, more patients in the TT group had brain metastases, which may explain the low OS of this group in our study. Previous studies reported similar findings (14).

Anna C Pavlick et al. (15), reported OS probabilities of 69% at one year, 60% at two years, and 58% at three years for their IT group; and 61% at one year, 37% at two years, and 27% at three years for their TT group. The PFS probabilities were 42% at one year and 35% at two years for IT vs. 32% at one year and 14% at two years for TT (15). The results of our study were similar to those of previous studies, with the IT group presenting a better two-year PFS and four-year OS.

In our study, the PFS of the TT group was 11.5 months, which was similar to the results of the COMBI-d study (2), while the PFS of the IT group in our study was 19.2 months, which was similar to the results of Checkmate 067. However, there were no statistical differences between the two groups (12).

In the ORR analysis, the TT group was significantly higher than the IT group (70.0% vs. 43.8%, p=0.04). In the COMBI-d and COMBI-v studies, the ORR for the D+T group was 69% and 64%, respectively (2, 4). Our results were similar to those previously reported. In the Checkmate 067 study, the ORR for NIV/IPI was 58%, however, in our study, the ORR for the IT group was lower. This may be due to our small sample size and because we only included patients with BRAF mutations, whereas 68% of the Checkmate 067 patients displayed BRAF wild-type.

The results of AEs in our study showed that the rates of AEs of grade ≥3 were 11.2% and 8% in the IT group and TT group, respectively (**Table 4**). This result is lower than the results in previously published studies. This may be due to significant improvements in IT and TT between 2011 and 2021, including the management of adverse reactions/reactions, dose, the duration of treatment, and combination. For example, patients with immune-mediated colitis can have their symptoms well controlled by the use of infliximab(16).

**Table 4:** Toxicity by treatment group.

AEs	IT		TT	
	Grade 1-2	Grade 3-4	Grade 1-2	Grade 3-4
diarrhea	10	2	6	1
nausea	2		2	
vomiting	2	1	1	
fatigue	7	3	6	2
fever	3		2	
AST/ALT increased	4	2	2	
leukopenia	1		1	
anemia	1		1	
rash	5	1	2	
hypertension	2		2	
Acute kidney injury	0		1	1

AEs, adverse events; IT, immunotherapy; TT, targeted therapy; AST, Aspartate aminotransferase; ALT, alanine aminotransferase.

Immunotherapy and targeted therapy protocols altered between 2011 and 2021; the sequencing of TT and IT was a hot topic in clinical research. A study on a mouse model of BRAF V600-mutant melanoma showed that the use of IT before TT maximized the antitumor efficacy (17). However, some real-world studies found that, compared with patients who received first-line immunotherapy, patients who received first-line targeted therapy had a higher proportion of liver metastases and abnormal lactate dehydrogenase, but there was no significant difference in survival between the two groups(18). Through our research results, we found that TT had a better ORR, but IT had a better OS and OS rate. This may be related to the “long tail effect” of immune checkpoint inhibitors, which is associated with the immune system’s ability to recognize and attack cancer cells, even after treatment has ended(19). This sustained immune response may be due to the development of long-lasting immunological memory, which allows the immune system to quickly recognize and attack cancer cells if they reappear. Oana-Diana Persa P et al. (20), reported that treatment with BRAF and MEK inhibitors usually results in a rapid response, but that this is often of limited duration. A durable, long-lasting response can be achieved with IT and may even enable an elective cessation of therapy, but latency to response is longer (21). In recent data published in the Journal of Clinical Oncology (9), the DREAMseq trial reported on 265 patients with advanced BRAFV600-mutant melanoma who received Step 1 therapy with either NIV/IPI (Arm A) or D/T (Arm B) and, at disease progression (PD), were enrolled in Step 2, receiving the alternate therapy, D/T (Arm C) or NIV/IPI (Arm D), respectively. Of patients with a median follow-up of 27.7 months, 27 had switched to Arm C and 46 to Arm D. ORR was: Arm A 46% (52/113), Arm B 43% (49/114), Arm C 48% (11/23), and Arm D 30% (8/27). Response rates were similar for the two step 1 regimens and for dabrafenib/trametinib, whether used in step 1 or step 2. In contrast, nivolumab/ipilimumab appeared to be less effective after disease progression with dabrafenib/trametinib as first-line therapy. There were 100 deaths (Arm A to C: 38; Arm B to D: 62). The two-year OS rate for those starting on Arm A was 72% (95% CI: 62-81%) and on Arm B 52% (95% CI: 42-62%) (log-rank p= 0.0095). PFS showed a trend in favor of Arm A (log-rank p=0.054). Both the PFS and OS curves had a biphasic pattern, with Arm B being above Arm A until 6 and 10 months, respectively. The treatment sequence beginning with the NIV/IPI combination resulted in superior OS, which became evident at 10 months, with longer Step 1 duration of response (DOR) and more ongoing responses than the treatment sequence beginning with D/T (22, 23). In a recent publication (8), the phase II SECOMBIT trial published its results on 251 patients selected for Arm A [Encorafenib+Binimetinib (E+B) until PD, followed by IT until PD], or Arm B (NIV/IPI until PD, followed by E+B until PD) or Arm C (E+B for eight weeks, followed by NIV/IPI until PD, followed by E+B until PD). The OS rate at two and three years was 65% and 54% in Arm A, 73% and 62% in Arm B and 69% and 60% in Arm C, respectively. Total PFS rate at two and three years was 46% and 41% in Arm A, 65% and 53% in Arm B, and 57% and 54% in Arm C, respectively. The OS and PFS rates at two and three years showed a better trend in Arms B and C (24). The treatment sequence for advanced BRAF-mutated melanoma still requires further clinical trials for verification and more mature data regarding OS.

Clinically, both the SECOMBIT (8) (although lacking the statistical power to show differences between arms) and DREAM-SEQ studies (9) advocate initiating treatment with the nivolumab and Ipilimumab combination in BRAF-mutated patients versus the combination of BRAF-MEK inhibitors. These clinical data are supported by various preclinical studies that observed that resistance to treatment with BRAF and MEKi causes an immunosuppressive microenvironment, wherein there is functional loss of CD103, dendritic cells, deficiency/exhaustion of CD8 T lymphocytes, and loss of antigen presentation. All this renders immunotherapy ineffective (25, 26). In contrast, murine models of melanoma treated primarily with IT followed by TT produced a prolonged tumor regression due to the accumulation of proinflammatory M1 macrophages, interferon Gamma secretion, and an increase in CD8 T lymphocytes (17).

In addition, several clinical trials evaluated the efficacy and safety of triple combination therapies. KEYNOTE−022, a phase II trial, comparing triple (pembrolizumab plus dabrafenib and trametinib) and double combination therapy (placebo with dabrafenib and trametinib), reported a median PFS of 16.9 months for triple combination therapies (95% CI 11.3–27.9) and 10.7 months (95% CI 7.2–16.8) for the doublet arm (HR 0.53; 95% CI 0.34–0.83). The median response duration was 25.1 months in the triplet arm vs. 12.1 months in the doublet arm (HR 0.32; 95% CI 0.17–0.59); grade 3–5 adverse events occurred in 70% of patients in the triplet and 45% in the doublet arm (27). The IMspire 150 trial compared the efficacy and safety of atezolizumab, vemurafenib, and cobimetinib (triplet arm) or atezolizumab placebo, vemurafenib, and cobimetinib (control arm). PFS was 10.6 months in the control arm and 15.1 months in the triplet arm; ORR was 66.3% and 65.0% in the triplet and control arms, respectively. The median DOR was longer in the triplet than in the control arm (21.0 months vs. 12.6 months). The rate of grade 3–4 adverse events was the same in both arms (79% in the triplet vs. 73% in the control arm) (28). Another combination was spartalizumab in combination with dabrafenib and trametinib. The primary endpoint was investigator-assessed PFS. The result was a median PFS of 16.2 months for the triplet combination vs. 12 months for the doublet combination (HR 0.82 [95% CI, 0.66 to 1.03]), thus the study was a negative study, not meeting its primary endpoint, however, it still contributes toward increasing the weight of evidence regarding these strategies (29).

Based on the results of the previous clinical trial, the combination of IT and TT had a better efficacy for PFS and ORR than double therapy or monotherapy, but there were more adverse events in the triple therapy. With the results of further clinical trials, triple combination therapies could provide more treatment options for melanoma.

Several studies have confirmed that, in the initial treatment of metastatic melanoma, the use of TT and IT combined has advantages, such as reducing the risk of toxicity and enhancing T-cell activity (30, 31). The mechanism is as follows: ① activation of the MAPK pathway in metastatic melanoma can promote the decline of tumor cell surface antigen expression such that they cannot be recognized by T cells, thus completing immune escape (32). BRAF and MEK targeted inhibitors can block the MAPK pathway, allowing the normal expression of tumor cell antigens for recognition by T cells (33); ② The BRAFV600 mutation promotes tumor cell immune escape, and the combined application of BRAF and MEK inhibitors can be used in the early stages to regulate the body’s immune microenvironment, trigger increased expression of melanoma cell surface antigens and CD8+T cytotoxicity markers (perforin and granzyme B), thereby promoting T cell infiltration and lymphocyte proliferation (34).

Furthermore, we evaluated the prognostic factors for advanced BRAF-mutated melanoma patients; we found that ECOG PS≥2, presence of liver metastases, and high LDH level from initiation of first-line treatment were all associated with differences in PFS and OS. Justin C. Moser et al., reported that age, ECOG, LDH, and treatment were associated with OS (35); Kaustav P. Shah et al., found that LDH level had the strongest association with both PFA and OS (36). Our results are consistent with these studies.

This study has several potential limitations mainly due to its retrospective nature; however, it reflects real-life daily clinical practice. In addition, the number of patients was small in some subgroups, potentially limiting the generalizability of the research findings. And the response to treatment was classified according to the RECIST 1.1. while RECIST 1.1 has been widely used in clinical trials and practice, it has certain limitations. These limitations include its reliance on the measurement of longest diameters of target lesions, the exclusion of functional imaging parameters like SUVmax, the lack of consideration for tumor burden, tumor mutation burden, and whole-body tumor volume. Considering these limitations and incorporating additional assessments, such as functional imaging or genomic profiling, may provide a more comprehensive evaluation of treatment response and improve our understanding of patient outcomes. Also, it is worth mentioning investigator bias, which could impact the treatment decision guided by patients’ symptoms and prognosis. We did not test the PDL1 status of the patients nor were adverse events evaluated; however, these points are also the subject of our ongoing investigations.

The reported trial results cited for comparison with the actual results shown in **Table 5**.

**Table 5:** The reported trial results cited for comparison with the actual results.

Clinical Trial	Treatment	ORR (%)	PFS (months)	OS (months)
Checkmate 067	Nivolumab+Ipilimumab vs Nivolumab vs Ipilimumab	NA	NA	72.1 vs 37.6 vs 19.9
COMBI-d+COMBI-v	Dabrafenib+ Trametinib	68%	11.1	25.9
ADMIRE	Dabrafenib + Trametinib or Vemurafenib + Cobimetinib	57.4%	9.2	22.6
Anna C Pavlick et al	BRAF + MEK inhibitors vs Nivolumab + Ipilimumab	NA	7.2 vs7.8	17.7 vs 48.4
DREAMseq trial	Nivolumab + Ipilimumab vs Dabrafenib +Trametinib	46% vs 43%	11.8 vs 8.8	NA
KEYNOTE-022	Pembrolizumab + Dabrafenib and Trametinib vs placebo with Dabrafenib and Trametinib	73% vs 67%	17.0 vs 9.9	46.3 vs 26.3
IMspire 150 trial	Atezolizumab, Vemurafenib, and Cobimetinib vs Atezolizumab placebo, Vemurafenib, and Cobimetinib	NA	15.1 vs 10.6	NA
BRAF mutated subgroup in this study	TT	70%	11.5	16.4
	IT	43.8%	19.2	33.4

ORR, objective response rate; PFS, progression-free survival; OS, overall survival; TT, targeted therapy; IT, immunotherapy; NA, Not Applicable.

## Conclusion

In this retrospective analysis, patients with advanced BRAF-mutated melanoma treated with first-line immunotherapy had a significantly longer PFS and OS than those treated with first-line BRAF/MEKi. However, first-line BRAF/MEKi treatment had a significantly higher ORR than first-line immunotherapy. Our results could guide the selection of first-line treatment according to the patient’s needs.

## Data availability statement

The raw data supporting the conclusions of this article will be made available by the authors, without undue reservation.

## Ethics statement

The studies involving humans were approved by Clinical Research Ethics Committee of Germans Trias i Pujol University Hospital. The studies were conducted in accordance with the local legislation and institutional requirements. The participants provided their written informed consent to participate in this study.

## Author contributions

CS: Conceptualization, Formal analysis, Funding acquisition, Methodology, Writing – original draft, Writing – review & editing. SE: Methodology, Writing – original draft, Writing – review & editing. NR: Writing – review & editing. CS-B: Writing – review & editing. AB: Writing – review & editing. AM-C: Writing – review & editing. AC: Writing – review & editing. ZL: Writing – review & editing. JM: Conceptualization, Methodology, Writing – original draft, Writing – review & editing.
